# From Photons to Behaviors: Neural Implementations of Visual Behaviors in *Drosophila*

**DOI:** 10.3389/fnins.2022.883640

**Published:** 2022-05-04

**Authors:** Leesun Ryu, Sung Yong Kim, Anmo J. Kim

**Affiliations:** ^1^Department of Electronic Engineering, Hanyang University, Seoul, South Korea; ^2^Department of Biomedical Engineering, Hanyang University, Seoul, South Korea

**Keywords:** *Drosophila*, Vision, Neural Circuits, Phototaxis, Optomotor response, Fixation, Navigation, Visual learning

## Abstract

Neural implementations of visual behaviors in *Drosophila* have been dissected intensively in the past couple of decades. The availability of premiere genetic toolkits, behavioral assays in tethered or freely moving conditions, and advances in connectomics have permitted the understanding of the physiological and anatomical details of the nervous system underlying complex visual behaviors. In this review, we describe recent advances on how various features of a visual scene are detected by the *Drosophila* visual system and how the neural circuits process these signals and elicit an appropriate behavioral response. Special emphasis was laid on the neural circuits that detect visual features such as brightness, color, local motion, optic flow, and translating or approaching visual objects, which would be important for behaviors such as phototaxis, optomotor response, attraction (or aversion) to moving objects, navigation, and visual learning. This review offers an integrative framework for how the fly brain detects visual features and orchestrates an appropriate behavioral response.

## Introduction

**FIGURE 1:**
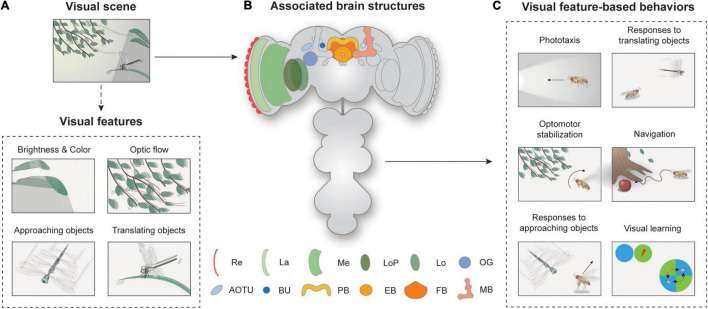
Overview of visual feature-based behaviors and associated brain structures. **(A)** Spatiotemporal features in visual scenes may induce appropriate behavior in walking or flying *Drosophila.* A visual scene consists of visual features, such as brightness, color, optic flow, and approaching or translating objects. **(B)** Neural structures for visual feature-based behaviors. Re: retina, La: lamina, Me: medulla, LoP: lobula plate, Lo: lobula, OG: optic glomeruli, AOTU: anterior optic tubercle, BU: bulb, PB: protocerebral bridge, EB: ellipsoid body, FB: fan-shaped body, MB: mushroom body. **(C)** Flies exhibit various visual feature-driven behaviors, including phototaxis, optomotor stabilization, responses to moving objects, and navigation and visual learning.

Animals with image-forming eyes, including humans and flies, distill visual features from their surrounding scene and use them to execute an appropriate action. A visual scene may contain multiple visual objects differing in their size, shape, brightness, color, position, and velocity, which we operationally define as visual features (Figure 1A). Neural circuits in the brain process the information from a visual scene to detect visual features, either from a single object (e.g., an approaching dragonfly) or from multiple objects that share the same feature (e.g., a group of leaves swaying synchronously), and then to induce an appropriate behavioral response. Among many visual systems, the *Drosophila* visual system has been arguably studied most intensively in the past few decades, especially at the level of neural circuits (Figure 1B).

How would one understand the *Drosophila* vision? David Marr and Tomaso Poggio proposed an influential theory, which stated that studies on information processing machines, either biological or artificial, should concern three levels of analyses: computations, algorithms, and implementations (Marr and Poggio, 1976; Marr, 1985). What is interesting is that this approach was inspired by a study of visually guided flight course control of house flies by Reichardt and Poggio (1976). The authors of that study attempted the three-levels-of-analysis approach but also acknowledged that their analyses primarily concerned the computational and algorithmic levels. The lack of data on the physiological and anatomical details of neural circuits at the time hampered their effort to understand the *Drosophila* vision “at a highly integrative level” (Reichardt and Poggio, 1975, 1976).

Since then, the understanding of *Drosophila* vision at the implementation level has progressed remarkably. The availability of premiere genetic toolkits, behavioral assays in tethered or freely moving conditions, and recent advances in connectomics have yielded an unprecedented level of understanding of the neural circuitry underlying the behavioral responses of *Drosophila* to visual cues (Jenett et al., 2012; Takemura et al., 2013; Zheng et al., 2018; Scheffer et al., 2020). The most intensively studied visual features perceived by *Drosophila* include brightness, color, optic flow, and translating or approaching movements of visual objects (Figure 1A). The behaviors associated with these features have also been well characterized (Figure 1C). Depending on the brightness or spectrum of the light, flies change their position or orientation, and this is called phototaxis. Optic flow is the whole-field visual motion of the surrounding visual features (Figure 1A) generated when an animal translates or rotates. The optic flow typically induces a corrective steering movement (Figure 1C). Approaching or translating objects could induce various behaviors, depending on the speed, position, and behavioral context of the animal. More complex visual behaviors include vision-based navigation and visual learning (Figure 1C).

Neural circuits involved in feature detection are found in the optic lobe and the central brain (Figure 1B). The retina consists of about 750 ommatidia covering 330° in azimuth and 180° in elevation, providing a wide-field vision to flies (Heisenberg and Wolf, 1984; Hardie, 1985). The light signals are then conveyed into the optic lobe, the largest visual structure in the *Drosophila* brain. The optic lobe comprises four major substructures: the lamina, medulla, lobula, and lobula plate. A recent study measured the total number of neurons in the optic lobe as around 100,000, comparable to that of the central brain, demonstrating the significance of vision in *Drosophila* as well as the complexity of visual processing (Raji and Potter, 2021). Visual projection neurons (VPNs) then carry this information to central brain structures, including the optic glomeruli (OG), the central complex (CX), and the mushroom bodies.

In this review paper, we describe recent advances in understanding how neural circuits in *Drosophila* are implemented to detect various features of a visual scene and to transform the visual feature further to execute an appropriate behavioral response. We organize each section by a specific visual feature-based behavior, from simpler to more complex features, with an emphasis on the associated neural circuits and their signaling mechanisms. This review will provide comprehensive, up-to-date knowledge on how neural circuits are implemented for major visual feature-based behaviors in *Drosophila*.

## Brightness, Color, and Phototaxis

**FIGURE 2:**
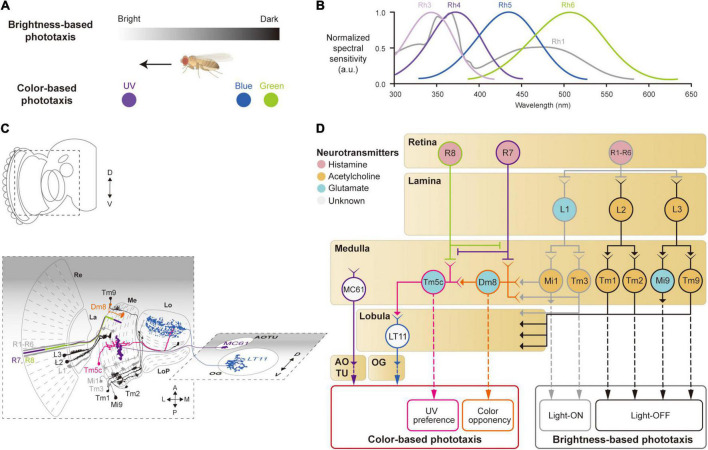
Neural circuits underlying the detection of brightness and colors for the phototaxis behavior. **(A)** *Drosophila* shows phototactic behaviors, seeking bright against dark (top) or ultraviolet (UV) against blue/green (bottom). **(B)** Spectral sensitivity curves of rhodopsins expressed in photoreceptor cells Graphical depiction is adapted from Hu and Stark (1977), Fischbach (1979), Gao et al. (2008), Yamaguchi et al. (2010), and Karuppudurai et al. (2014). **(C)** Schematic illustration of the structures and neurons described in panel **(D)**. Images of neurons are adapted from Fischbach and Dittrich (1989) and Otsuna et al. (2014). Re: retina, La: lamina, Me: medulla, LoP: lobula plate, Lo: lobula, OG: optic glomeruli, AOTU: anterior optic tubercle. **(D)** Schematic of a neural circuit related to the phototaxis. Photoreceptor cells (R1 – R6) detect changes in brightness and pass light-ON (gray outlines) or OFF (black outlines) information to downstream neurons. R7 and R8 cells respond to UV light (purple and green outlines) and convey the information to different neural pathways for color-based phototaxis (orange, pink and blue outlines), which include color opponency (orange outline) and UV preference (pink outline). The face color of a circle represents the type of neurotransmitters released by a neuron, and the line color of a circle matches that of the neuron in panel **(C)**.

After redirecting his interest from molecular biology to behavioral genetics, Seymour Benzer noted the fly’s relentless effort to move toward the window—phototactic behavior—and identified the first phototaxis mutant (Benzer, 1967). The strength of the phototactic response depends not only on the intensity but also on the color of the light (Bertholf, 1932; Hadler, 1964; Schümperli, 1973; Otsuna et al., 2014; Figure 2A). When flies encounter areas with different colors of light, they are most strongly attracted to ultraviolet (UV) light (Hu and Stark, 1977; Fischbach, 1979; Gao et al., 2008; Yamaguchi et al., 2010; Karuppudurai et al., 2014; Figure 2A). This color preference, however, was shown to vary according to a circadian rhythm (Hu and Stark, 1977; Lazopulo et al., 2019). Female flies were shown to be more strongly attracted to males with vivid colored wings than those with dull wings (Katayama et al., 2014).

Neurons involved in brightness- and color-based phototactic behaviors have been identified in various visual structures, from the retina to the OG (Hadler, 1964; Stark et al., 1976; Otsuna et al., 2014; Strother et al., 2014; Timaeus et al., 2020; Figure 2C). First, photoreceptor cells (R1–R8) in the retina convert the energy of photons into neural signals by depolarizing in response to increments in light intensity (light-ON hereafter), unlike mammalian photoreceptor cells that hyperpolarize to light (Hardie, 1989; Hardie and Raghu, 2001). R1–R8 cells express distinct light-sensitive G-protein-coupled receptors, rhodopsins, Rh1 and Rh3–6, that have varying spectral sensitivities (Salcedo et al., 1999; Figure 2B). The R1–R6 cells express the same rhodopsin (Rh1) and respond to a wide range of light, showing a bimodal spectral tuning peaking in the green and UV spectra. R7 and R8 cells show different spectral tuning properties according to the type of rhodopsin they express stochastically: about 70% of the R7 cells express Rh4; the remaining R7 cells express Rh3. Similarly, the R8 cells express either Rh5 or Rh6. All the photoreceptor cells (R1–R8) are housed in a single, isolated optical unit, called an ommatidium (Stark et al., 1976; Kirschfeld et al., 1977; O’Tousa et al., 1985; Fortini and Rubin, 1990; Rister and Desplan, 2011).

When depolarized by a light-ON stimulus, the photoreceptor cells in *Drosophila* release histamine, unlike glutamate in the mammalian photoreceptor cells, to postsynaptic neurons in the subsequent visual structures: the lamina for R1–R6, and the medulla for R7 and R8 (Fischbach and Dittrich, 1989; Meinertzhagen and O’Neil, 1991; Hardie and Raghu, 2001; Takemura et al., 2013; Figures 2C,D). In the lamina, lamina monopolar cells (LMCs), L1–L5, receive light signals from the R1–R6 cells *via* histamine-gated chloride channels encoded by the gene *ort*, which cause them to hyperpolarize to a light-ON stimulus (Gengs et al., 2002; Rister et al., 2007; Gao et al., 2008; Figures 2C,D). Each LMC receives inputs from multiple photoreceptor cells in neighboring ommatidia pointing at the same coordinates in the visual space (Braitenberg, 1967; Meinertzhagen and O’Neil, 1991). This is called neural superposition and is crucial for enhancing the signal-to-noise ratio in vision (de Ruyter van Steveninck and Bialek, 1995). Studies have shown that the responses of LMCs differ in their dynamics: L1 and L2 cells show highly transient responses to the light-ON stimulus, whereas L3 cells exhibit persistent responses, likely providing luminescence information from the environment (Rister et al., 2007; Silies et al., 2013; Strother et al., 2014; Fisher et al., 2015; Yang et al., 2016; Ketkar et al., 2020).

L1–L3 cells project axons to distinct medulla layers (L1 to M1/M5, L2 to M2, and L3 to M3) and release different types of neurotransmitters. L1 cells release glutamate and hyperpolarize medulla intrinsic 1 (Mi1) and transmedullary 3 (Tm3) neurons *via* GluClα, a glutamate-gated chloride channel (Yang et al., 2016; Strother et al., 2017; Molina-Obando et al., 2019; Davis et al., 2020; Figure 2D). By contrast, L2 and L3 cells release acetylcholine to depolarize postsynaptic neurons, Tm1/Tm2 and Tm9/Mi9 (Fisher et al., 2015; Yang et al., 2016; Davis et al., 2020; Ramos-Traslosheros and Silies, 2021; Figure 2D, black lines). This leads to two visual pathways with opposing visual responses: hyperpolarization of L1, L2, and L3 cells, in response to a light-ON stimulus, leads to depolarization of Mi1/Tm3 cells (thus called light-ON neurons), but hyperpolarization of Tm1/Tm2 and Tm9/Mi9 cells (light-OFF neurons). These light-ON/light-OFF circuits are important for most visual behaviors, including phototaxis.

Color-based phototaxis starts from R7 and R8 cells (Figures 2B,D). R7 is most strongly sensitive to long-UV/short-UV, whereas R8 is sensitive to blue/green, depending on the types of rhodopsin they express (Montell et al., 1987; Chou et al., 1996; Papatsenko et al., 1997; Schnaitmann et al., 2018). R7 and R8 project directly to the medulla layers, bypassing the lamina (R7 to M6 and R8 to M4) (Fischbach and Dittrich, 1989; Gao et al., 2008; Takemura et al., 2013; Schnaitmann et al., 2018; Figures 2C,D). In association with their different spectral properties, it has been found that R7 and R8 form direct synaptic contacts in the M1−M3 layers to inhibit each other *via* a HisCl1 (histamine-gated chloride channel 1), contributing to enhanced color discrimination (Schnaitmann et al., 2018; Figure 2D).

The UV information from R7 is also combined with signals from the R1–R6 cells in a class of medullar neurons called distal medulla 8 (Dm8) (Karuppudurai et al., 2014; Li et al., 2021; Pagni et al., 2021), which is required for the UV preference behavior (Gao et al., 2008; Karuppudurai et al., 2014). Dm8 receives hyperpolarizing input from R7 *via* Ort receptors and depolarizing input from Mi1/Tm3 cells *via* cholinergic receptors, whose light responses are derived from R1–R6 (Li et al., 2021; Pagni et al., 2021). Consequently, Dm8 neurons hyperpolarize to UV light and depolarize to blue/green light. The color opponent signals from Dm8 are then further passed *via* glutamatergic synapses to another medulla neuron, Tm5c (Karuppudurai et al., 2014; Figure 2C). Tm5c also receives input from R8 *via* Ort receptors and is shown to be important for green phototaxis (Gao et al., 2008; Karuppudurai et al., 2014; Figure 2C).

The color information in the optic lobe is conveyed to the central brain *via* VPNs, such as lobula tangential 11 (LT11) neurons and medulla columnar 61 (MC61) neurons (Otsuna et al., 2014; Lin et al., 2016; Timaeus et al., 2020). LT11 cells respond to blue light and project to the posterior ventral lateral protocerebrum (PVLP), whereas MC61 cells respond to green/UV light and project to the anterior optic tubercle (AOTU) (Figures 2C,D). LT11 dendrites form synaptic connections with Tm5c axons in lobula layers, Lo4 to Lo6 (Otsuna and Ito, 2006; Lin et al., 2016). The direct synaptic connection between Tm5c and LT11 indicates that the R8→Tm5c→LT11 pathway likely mediates blue-specific phototaxis. MC61, also known as medullo-tubercular neurons, conveys visual information from the medulla (M2, M6, and M8 layers) to the AOTU (Otsuna et al., 2014; Panser et al., 2016; Timaeus et al., 2020). Although MC61 shows similar spectral preferences to Dm8, they do not appear to form direct synaptic contacts with each other. Instead, the expression of *ort* in MC61 suggests that MC61 is likely to receive color features directly from photoreceptor cells such as R7 (Gao et al., 2008; Timaeus et al., 2020; Figure 2D). Together, these findings show that color features are conveyed to the central brain through VPNs for color-based phototaxis.

The *Drosophila* visual system consists of largely parallel and independent visual pathways, such as the light-ON, the light-OFF, and the color processing pathways, as in the mammalian visual system. However, recent studies have also pointed out that these pathways interact with each other at various levels. For example, R6 and R8 neurons interact *via* gap junctions, which causes light signals from R8 to enter L1, L2, and L3, and eventually influences behaviors associated with the optic flow (Wardill et al., 2012). On the contrary, deletion of *rh1* in R1−R6 leads to defects in color preference to UV or blue (Yamaguchi et al., 2010). Furthermore, downstream of R8, Tm5c receives synaptic inputs from L3, whose functions have not yet been identified (Gao et al., 2008). In summary, these results suggest crosstalk between the color and brightness pathways, but further studies are needed to understand the role and detailed mechanisms of this communication. In addition, studies on central and descending pathways are required for a complete understanding of the neural implementations of phototactic behaviors.

## Motion Detection and Optomotor Responses

**FIGURE 3:**
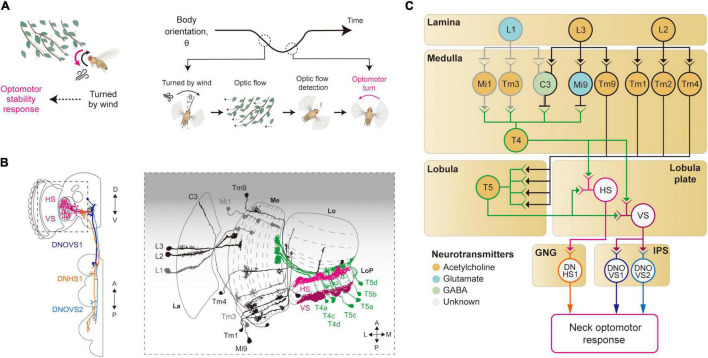
Neural circuits underlying motion detection and optomotor response. **(A)** When *Drosophila* is turned by a gust of wind, it recovers its heading immediately. In this reflex, the rotational whole-field visual motion, called optic flow, is detected by the visual system and induces the so-called optomotor response. **(B)** Schematic illustration of the neurons related to the motion detection and the optomotor response. The graphical depiction is adapted from Suver et al. (2016), Fischbach and Dittrich (1989), and Namiki et al. (2018). The colors of neurons match those in panel **(C)**. La: lamina, Me: medulla, LoP: lobula plate, Lo: lobula. **(C)** Schematic diagram of neural circuits related to motion detection and optomotor response. T4 and T5 cells receive visual inputs from light-ON (gray outline) and light-OFF (black outline) pathways and generate motion-sensitive signals with direction sensitivity. Subsequent integration of local motion signals in T4 and T5 cells occurs in the lobula plate tangential neurons, such as HS and VS (pink and purple outlines), to give rise to optic flow signals. These signals are transmitted to descending neurons, such as DNHS1, DNOVS1 and DNOVS2 (orange, dark blue and light blue outlines), leading to the optomotor response. The face color of a circle represents the type of neurotransmitters released by a neuron, and the line color of a circle matches that of the neuron in panel **(B)**. GNG: gnathal ganglia, IPS: inferior posterior slope.

For its light body weight and relatively large wing size, flying *Drosophila* are prone to deviate from their intended course, even in a low-turbulence wind. Each time a fly rotates or translates due to a gust of wind, its eyes will experience the visual motion of the surrounding scene that will undesirably blur its vision. To minimize the duration of such blurs and also to maintain the intended flight course, flies perform robust stability reflexes using their vision and other sensory modalities (Sherman and Dickinson, 2004; van Breugel and Dickinson, 2012; Muijres et al., 2014). Namely, when a whole-field visual motion (optic flow) is sensed, they perform robust corrective flight (or walking) maneuvers, called the optomotor response (Mauss and Borst, 2020; Figure 3A).

Studies of optomotor behavior and the underlying neural implementation have a long history in *Drosophila* neuroscience. The first report on the optomotor response dates back to 1934, with freely walking flies (Hecht and Wald, 1934). Subsequent research quickly sought to uncover the genetic bases of the optomotor response using mutant screening techniques (Kalmus, 1943; Götz, 1964, 1970). Notably, Götz (1970) devised a countercurrent assay for this behavior, as Seymour Benzer did for phototaxis (Benzer, 1967). In his paper, Götz stated that the study of the optomotor response requires at least three neural algorithms to be understood: (1) sensing local motion by comparing light intensity changes across adjacent ommatidia, (2) integrating local motion across retina to detect optic flow, and (3) passing this information to appropriate motor programs.

### Local Motion Detection

Local motion detection is a prerequisite for the optomotor response, and thus studies of the optomotor response naturally led to the studies on how local motion is computed from visual images sensed in the retina. In particular, pivotal works by Hassenstein and Reichardt on how walking beetles choose their courses in response to different motion-like visual patterns laid out the framework for the computation of local motion (Hassenstein, 1951; Hassenstein and Reichardt, 1956). That is, they proposed a correlative model of motion computation, the so-called Hassenstein–Reichardt motion detector. In this model, the local motion is computed in two steps: delaying a light signal from an ommatidium and multiplying it with a less delayed signal from an adjacent ommatidium. Since the proposal of this model, studies of *Drosophila* motion detectors have been a process of identifying neural correlates of building blocks in the model.

The first neurons that exhibit motion sensitivity along the visual pathway are the T4 cells in the medulla and T5 cells in the lobula (Figures 3B,C). This leads to the main question: How do these cells compute motion signals from the non-motion signals they receive from the preceding visual neurons? Blocking L1 and L2 cells in the lamina—either by mutating their Ort receptors or blocking the synaptic release—led to an impairment in the optomotor response, suggesting that these lamina cells provide major visual inputs to T4/T5 cells (Rister et al., 2007; Gao et al., 2008; Joesch et al., 2008; Joesch, 2009; Strother et al., 2014). Postsynaptic neurons to the lamina cells—Mi1 and Tm3 cells receiving inputs from L1 cells (light-ON pathway), and Mi9 and Tm9 cells receiving inputs from L3 cells (light-OFF pathway)—show responses to light intensity changes, but still no motion sensitivity (Fisher et al., 2015; Strother et al., 2017). However, these cells showed distinct time delays that can be in theory used to compute the motion signals observed in T4/T5 cells (Behnia et al., 2014; Gruntman et al., 2018; Shinomiya et al., 2019). Specifically, some neurons in the light-ON pathway, such as Mi4 and Mi9, carry the time-delayed visual signals to T4 cells, and neurons in the light-OFF pathway, such as Tm9 and CT1, pass the signals to T5 cells (Figures 3B,C). When these delayed signals are compared with non-delayed inputs (Mi1 and Tm3 for the light-ON pathway, and Tm1–4 for the light-OFF pathway), a spatiotemporally correlated change in local visual inputs causes T4/T5 cells to depolarize, as in the Hassenstein-Reichardt model. However, these studies eventually led to a new motion detection model to reflect the anatomical and physiological data faithfully (Gruntman et al., 2018; Shinomiya et al., 2019). In the new model, the local motion is calculated from three inputs (instead of the two): non-delayed central input compared with−by subtraction followed by division−the delayed inputs from the two adjacent columns.

### Optic Flow Detection and Optomotor Response

Once the local motion is detected, this information can be used downstream to detect more complex visual features. In particular, an optic flow pattern is detected by integrating inputs from T4/T5 cells for a large visual field, which will eventually lead to the optomotor response. The existence of optomotor response-mediating neurons was first demonstrated by a *Drosophila* mutant named optomotor-blind*^H31^* (*omb^H31^*) that showed a highly compromised optomotor response (Heisenberg and Götz, 1975). In *omb^H31^* flies, lobula plate tangential cells (LPTCs) including horizontal system/vertical system (HS/VS) cells exhibit developmental defects, an observation that supports their roles in the optomotor response (Heisenberg and Götz, 1975; Heisenberg et al., 1978). Most recent studies used highly specific GAL4 driver lines for HS/VS cells and confirmed that optogenetic activation of these cells induced turning responses both in flight and in walking, providing causal evidence for their role (Haikala et al., 2013; Fujiwara et al., 2017; Busch et al., 2018).

The visual properties of HS/VS cells were thoroughly examined by electrophysiology experiments, first in *Calliphora* and then in *Drosophila* (Krapp et al., 1998; Joesch et al., 2008; Schnell et al., 2010). Those studies demonstrated that the receptive fields of HS/VS cells are precisely matched to an optic flow associated with self-rotation around distinct rotation axes, therefore suited to control the optomotor response (Krapp et al., 1998). That is, HS cells respond precisely to yaw-associated optic flow, VS1-3 cells to pitch-associate optic flow, and VS4-6 cells to roll-associated optic flow (Krapp et al., 1998; Joesch, 2009). The high precision of the HS/VS cell receptive field for the optic flow detection arises because of its dendritic innervation pattern in the lobula plate. The elementary motion detectors, the T4/T5 cells, project to the four distinct layers of the lobula plate, according to their preferred direction of motion and with a retinotopic organization (Maisak et al., 2013). The dendrites of the HS/VS cells then receive direct synaptic input from T4/T5 cells across the lobula plate, pooling local motion information from a large visual space for a specific direction (Suver et al., 2016; Boergens et al., 2018; Figures 3B,C). HS/VS cells depolarize in response to a motion in the preferred direction and hyperpolarize in response to the non-preferred direction. The depolarizing response is provided directly from cholinergic T4/T5 cells from the layer that HS/VS cells innervate (Maisak et al., 2013; Mauss et al., 2014, 2015), whereas the hyperpolarizing inputs arrive indirectly from an adjacent lobula plate layer *via* a set of glutamatergic interneurons, such as the lobula plate intrinsic (LPi) neurons LPi3-4 and LPi4-3.

How do HS/VS cells activate the motor system for the stability reflex? HS/VS neurons are shown to connect directly to the descending neurons (DNs) DNHS1 (descending neuron of the horizontal system 1, also called DNp15) and DNOVS1 (descending neuron of the ocellar and vertical system 1, also called DNp20) in the gnathal ganglia (GNG) and the inferior posterior slope (IPS) (Suver et al., 2016; Namiki et al., 2018; Figures 3B,C). Then, the axons of DNHS1/DNOVS1 cells terminate in the prothoracic region in the ventral nerve cord (VNC) to eventually control neck muscles and then the head movement. In line with these anatomical observations, a recent silencing experiment verified that the HS cells are important for the head optomotor response, but less so for the wing optomotor response, at least in flight (Kim A. J. et al., 2017). This suggested the existence of yet-unidentified visual pathways that mediate the wing optomotor response, perhaps a pathway complementary to that of the HS/VS cells.

Collectively, these studies identified neural circuits involved in local motion detection, as well as the optomotor response. However, some important questions remain unanswered. First, what are the synaptic and dendritic mechanisms of motion computation in T4/T5 cells? Although different models have been suggested for the dendritic computation of these cells, the specific molecular mechanisms remain to be elucidated. Second, HS/VS cells appear to regulate head optomotor responses but contribute only weakly, if at all, to wing optomotor responses, as mentioned above. Neurons that regulate the stabilization motion of the wings and some other body parts during optomotor responses remain to be identified. Furthermore, a series of studies have reported that HS/VS cells are systematically modulated by locomotive actions in flight and walking to suppress self-generated visual feedback inputs (Kim et al., 2015; Kim A. J. et al., 2017; Fujiwara et al., 2017; Fenk et al., 2021). The neural circuits that carry the motor-related inputs to these neurons also remain to be identified.

## Moving Objects and Associated Behaviors

**FIGURE 4:**
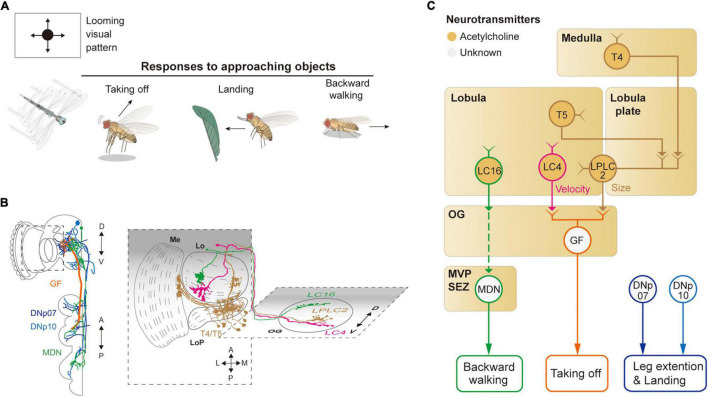
Neural circuits underlying avoidance behavior to approaching (looming) visual objects. **(A)** Approaching visual objects may induce distinct behaviors, such as take-off, landing, and backward walking, depending on their size and velocity. **(B)** Schematic illustration of neurons related to the loom avoidance behavior. Graphical depiction is adapted from Sen et al. (2017), Namiki et al. (2018), Wu et al. (2016), and Feng et al. (2020). The colors of neurons match those in panel **(C)**. Me: medulla, LoP: lobula plate, Lo: lobula, OG: optic glomeruli. **(C)** Schematic diagram of neural circuits related to loom-induced avoidance behavior. In optic glomeruli, LPLC2, LC4, LC16 VPNs are shown to be sensitive to a looming visual pattern. LPLC2 cells integrate signals from T4/T5 cells in the lobula plate to detect approaching objects (ocher outline). LC4 cells respond to the looming pattern with their dendrites in lobula (pink outline). LPLC2 and LC4 cells pass these signals to giant fibers (GFs), which induce the take-off response (orange outline). Approaching objects may also cause the landing response *via* DNs, DNp07, DNp10 (blue outline). Moonwalker descending neurons (MDN) (green outline) receive the visual information from LC16 cells and induce backward walking. The face color of a circle represents the type of neurotransmitters released by a neuron, and the line color of a circle matches that of the neuron in panel **(B)**. OG: optic glomeruli, MVP: medial ventral protocerebrum, SEZ: subesophageal zone.

**FIGURE 5:**
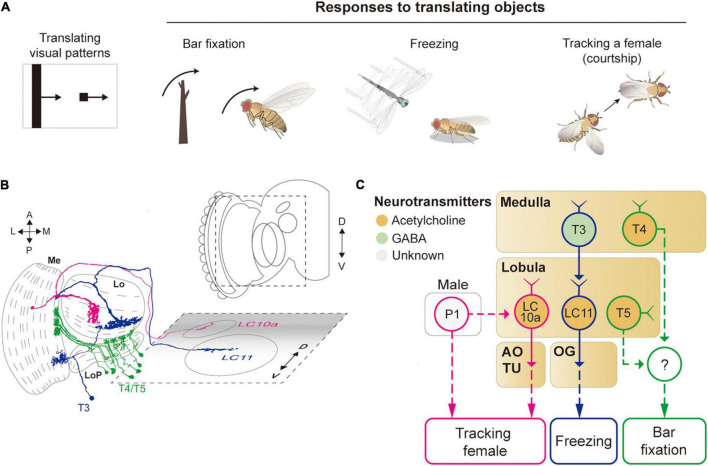
Neural circuits underlying the detection of translating object for object-related behaviors. **(A)** Translating objects include a moving bar and a small spot (left pattern). Flying flies show robust attraction to a moving bar, whereas a moving spot causes freezing in walking flies and chasing by aroused males. **(B)** Schematic illustration of neurons related to the translating object-induced behavior. Graphical depiction is adapted from Fischbach and Dittrich (1989) and Wu et al. (2016). The colors of neurons match those in panel **(C)**. Me: medulla, LoP: lobula plate, Lo: lobula, OG: optic glomeruli, AOTU: anterior optic tubercle. **(C)** Schematic diagram of neural circuits related to the moving object-induced behaviors. Bar attraction is dependent on T4/T5 local motion detections (green outline). LC11 is highly selective to a moving, dark spot and is important for spot-induced freezing behavior (blue outline). LC10 mediates spot-induced chasing behavior in male flies when sexually aroused (pink outline). The face color of a circle represents the type of neurotransmitters released by a neuron, and the line color of a circle matches that of the neuron in panel **(B)**. OG: optic glomeruli, AOTU: anterior optic tubercle.

Vision endows an animal with the ability to sense moving objects from afar. The movement of an object can be decomposed into a translational (or tangential) and a radial component from the fly’s perspective. If the motion features of a moving object collectively indicate imminent danger, they will induce actions such as freezing, jumping, backward walking, and even flight take-off in some insects, depending on the behavioral context being faced. On the contrary, if the motion features of an object indicate potential opportunities, such as food or mating partners, animals will turn toward or even chase the object (Figures 4A, 5A). In the following, we will first discuss the avoidance of an approaching object, then the attraction to or avoidance of a translating object.

Detection of a moving object, or detection of a static object by a moving animal, has been studied mostly at the level of VPNs, which relay visual signals from the optic lobe to the central brain regions. Four major types of VPNs are named by the position and shape of their dendrites: MCs, LCs for lobula columnar neurons, LPLCs for lobula plate and lobula columnar neurons, and LPTCs. The dendrites of these cells innervate a subset of ∼750 retinotopically arranged columns in each structure. The axons of these neurons mainly target a glomerular structure in the PVLP and AOTU, forming approximately 20 discrete neuropils termed OG. Thus, VPNs act to reduce the dimension of visual information from ∼750 to ∼20 (Panser et al., 2016; Wu et al., 2016; Davis et al., 2020). This dimensional reduction is why the OGs are considered as candidate structures for the detection of visual features essential for the survival of the animal.

### Avoidance of an Approaching Object

When confronted with a fast-approaching object, such as a fly swatter, resting or walking flies execute a rapid, stereotyped take-off action (Figure 4A). The take-off action consists of a rapid extension of the midleg and depression of the wings, a mechanism that is shown to be mediated by a pair of large DNs, known as giant fiber neurons (also called DNp01) (Bacon and Strausfeld, 1986; von Reyn et al., 2014; Figures 4B,C). DNp01 receives visual information mostly from LPLC2 and LC4 VPNs. Although both LPLC2 and LC4 are shown to be sensitive to a looming visual pattern, LPLC2 is sensitive to the terminal size of the looming pattern, whereas LC4 is sensitive to the velocity of the pattern (Ache et al., 2019b). LPLC2 neurons receive some of their visual inputs from T4/T5 cells, and their dendrites in the lobula plate are shown to be organized to selectively detect a radially expanding visual pattern (Klapoetke et al., 2017). LPLC2 and LC4 are connected *via* cholinergic synapses to DNp01 to induce jumping behaviors in the face of a fast-approaching object.

To a slowly approaching object, walking flies are shown to move backward. Known to induce backward walking, the moonwalker descending neurons (MDNs) receive their visual inputs from LC16 VPNs (Bidaye et al., 2014; Sen et al., 2017; Figures 4B,C). LC16 is responsive to an approaching visual object, akin to LPLC2 and LC4, but their activation leads to backward walking instead of jumping (Wu et al., 2016; Sen et al., 2017). Despite the lack of direct contact between LC16 and MDNs, they are shown to be functionally connected *via* a pathway comprising at least one cholinergic synapse (Sen et al., 2017).

In response to a looming visual pattern, flies in flight show landing or escape maneuvers. The landing response is characterized by the leg extension and is controlled by two DNs: DNp07 and DNp10 (Ache et al., 2019a; Figures 4B,C). These DNs innervate some OGs with their dendrites and project to leg neuropils in the VNC. Light microscopic and electron microscopic observations showed that these DNs receive visual inputs from LPLC3 and LPLC4 VPNs (Namiki et al., 2018). However, the visual properties of these VPNs are yet to be characterized. Likewise, the visuomotor circuit responsible for the avoidance flight maneuvers in response to a laterally (or even centrally) looming visual pattern is yet to be identified.

### Attraction to or Avoidance of Translating Objects

A translating object may trigger divergent behaviors, depending on its shape and the behavioral state of the animal (Figure 5A). For example, a tethered, flying *Drosophila* exhibits robust fixation to a dark vertical bar, menotaxis to a bright spot, and anti-fixation to a dark spot (Reichardt and Wenking, 1969; Maimon et al., 2008; Giraldo et al., 2018). In walking flies, the fixation to a vertical bar was observed, but not the avoidance to a small translating spot (Bahl et al., 2013; Giraldo et al., 2018). When a male fly is sexually aroused, it chases a female or similar objects robustly (Agrawal et al., 2014; Kohatsu and Yamamoto, 2015).

The attraction to a vertical bar is thought to be due to its visual resemblance to trees, a major feeding site for fruit flies. However, the neural circuits that mediate the bar fixation have only partially been understood. The detection of the motion of a translating bar was once thought to require the elementary motion detectors, T4/T5 cells, but blocking these cells affected the bar fixation only mildly or only for a specific translation velocity (Bahl et al., 2013; Fenk et al., 2014). Although one type of VPN cell (LC15) was shown to be sensitive to a moving bar with high selectivity, their inactivation did not notably change the bar attraction behavior (Städele et al., 2020).

For small spots, walking *Drosophila* may induce either freezing or avoidance behaviors. Two types of LC neurons, LC11 and LC10, are associated with these behaviors (Figures 5B,C). In particular, LC11 cells are shown to be sensitive to small dark spots and required for spot-induced freezing behavior (Keleş and Frye, 2017; Tanaka and Clark, 2020). Furthermore, these cells are important for sensing the movement of nearby conspecifics and thereby regulating the freezing behavior (Ferreira and Moita, 2020). The dendrites of LC11 cells receive inputs from T3 cells through GABAergic synapses. However, it is unclear how the hypercomplex properties of LC11 cells arise at this point. Moreover, the downstream pathway from LC11 to the central brain and motor systems leading to the freezing behavior is not yet understood. In flight, flies are shown to strongly avoid a small dark spot, but a neural circuit underlying this behavior remains to be identified (Maimon et al., 2008).

As mentioned above, when sexually aroused, male flies chase females robustly. In this behavior, visual information about the target female is detected by one subtype of LC10 cells, LC10a, that project to AOTU (Ribeiro et al., 2018; Sten et al., 2021). Optogenetic activation of LC10a neurons not only generates tracking behaviors but also induces wing extension in male flies (Figures 5B,C). Male-specific P1 neurons (integrate chemosensory cues) are essential to control the arousal state and courtship behavior (Kohatsu and Yamamoto, 2015). Recently, P1 cells were found to gate the visual signaling in LC10a and eventually increase tracking behaviors (Sten et al., 2021), but because P1 is not directly connected with LC10a, additional neurons remain to be identified to bridge the gap between these cell types.

Visual features detected at the level of the OG are used to command various visual behaviors. For example, several types of DNs are connected directly to VPNs in some OGs, such as DNp01, DNp07, and DNp10 (Namiki et al., 2018; Ache et al., 2019a). These neurons convey visual feature information directly to the VNC, in which central pattern generators coordinate motor movements for various behaviors. In addition, some OGs, albeit not connected directly to DNs, are shown to induce specific motor programs when experimentally activated, which suggests a substantial impact of VPNs on motor actions (Sen et al., 2017).

Overall, *Drosophila* show a variety of behaviors in response to moving objects. To date, the visual features of a moving object have been identified mostly in VPNs in the OG and AOTU. The visual signals are transmitted from the OG and AOTU to DNs directly or indirectly and eventually lead to associated motor outputs. Interestingly, multiple OGs were shown to represent the same visual features, akin to olfactory glomeruli (Wu et al., 2016; Ache et al., 2019b; Städele et al., 2020). Thus, it remains to be studied how the same visual feature represented by multiple visual structures are combined to lead to a specific action. A recent study demonstrated a topographic map of visual feature sensitivity in OG, at least for a subset of glomeruli (Klapoetke et al., 2022). Studies on the functions of OGs and behaviors have a relatively short history; therefore, it is expected that more interesting roles of OGs will be revealed in future studies.

## Vision-Based Spatial Navigation

**FIGURE 6:**
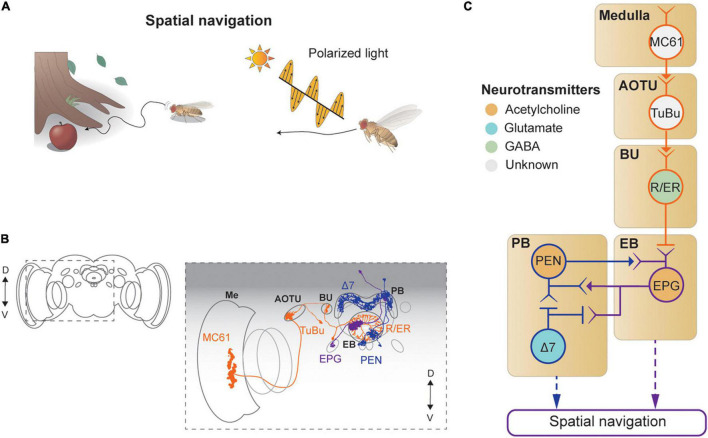
Neural circuits underlying visually-guided spatial navigation. **(A)** *Drosophila* navigates environments based on visual landmarks, sun position, and polarization. **(B)** Schematic illustration of structures of neurons described in panel (C), adapted from images in Omoto et al. (2017) and Hulse et al. (2021). Me: medulla, AOTU: anterior optic tubercle, BU: bulb, PB: protocerebral bridge, EB: ellipsoid body. **(C)** A schematic diagram of neural circuits involved in navigation and visual learning. The face color of a circle represents the type of neurotransmitters, and the line colors match those in panel **(B)**.

*Drosophila* shows sophisticated spatial navigation behavior, and vision provides key sensory cues for navigation. Walking or flying *Drosophila* use visual features, such as the surrounding landscape, sun position, and polarization, to determine their navigation course (Ofstad et al., 2011; Giraldo et al., 2018; Warren et al., 2018; Hardcastle et al., 2021; Figure 6A). Flies learn to associate visual landscape with the current position and heading so as to remember the location of food or safe zone (Ofstad et al., 2011; Kim and Dickinson, 2017). The heading direction can also be influenced by the polarization of light (Weir and Dickinson, 2012; Hardcastle et al., 2021) or the angle of the sun (Giraldo et al., 2018). Furthermore, flies perform local search behavior after encountering a food site (Kim and Dickinson, 2017; Behbahani et al., 2021). This behavior appears to involve path integration through the computation of the internal sense of the position and the orientation of the animal (Kim and Dickinson, 2017). Correspondingly, neural structures in the CX−ellipsoid body (EB), protocerebral bridge (PB), noduli, and fan-shaped body−were shown to be important for calculating and sustaining these navigation signals based on visual inputs (Wolff et al., 2014; Figures 1B, 6B).

Visual signals enter the CX *via* the anterior visual pathway (Omoto et al., 2017; Figures 6B,C). First, a class of medulla neurons (MC61) passes visual signals to the AOTU, which are then carried, *via* tuberculo-bulbar neurons, to a structure called the bulb, a major input structure to the CX. Next, a set of neurons with ring-shaped axons, thus called ring neurons, receive visual signals from the tuberculo-bulbar neurons *via* cholinergic synapses. The ring neurons show a center-surround receptive field, like simple cells in the mammalian visual cortex, and transmit information to the EB (Seelig and Jayaraman, 2013). Ellipsoid body projection (EPG) neurons receive the heading input in one of 16 compartments that form the EB, based on the visual inputs from ring neurons (Seelig and Jayaraman, 2015; Green et al., 2017; Kim S. S. et al., 2017). Synapses between the ring neurons and EPG are GABAergic and subject to strong Hebbian-type plasticity, by which the dynamic visual landscape is mapped to a heading signal (Kim S. S. et al., 2017; Xie et al., 2017; Fisher et al., 2019; Kim et al., 2019; Figure 6C).

This heading signal network consists of multiple cell types in the EB and PB, forming a recursive network that maintains the stability of the heading signal while moving it according to internal and external cues indicating self-rotation. Specifically, EPG neurons transmit the signal from the EB to the PB (as well as to the gall), and the PB–EB–noduli (PEN) and PB–EB–gall (PEG) neurons connect in the reverse direction, both *via* cholinergic synapses (Turner-Evans et al., 2020; Figures 6B,C). As a result of this structure, the heading signal in this network is maintained even in the dark (Seelig and Jayaraman, 2015; Green et al., 2017).

A class of PB local neurons, called Δ7 neurons (“Δ7” refers to the 7-glomerulus spacing between axonal terminals in single cells of this anatomical class), implement mutual inhibition *via* glutamatergic synapses for the heading signal within the PB (Franconville et al., 2018; Turner-Evans et al., 2020; Figures 6B,C). In particular, Δ7 cells receive synaptic inputs from the EPG and produce inhibitory signals to EPG and PEN cells. The mutually inhibitory connections between EPGs *via*Δ7 cells appear to be important for maintaining the heading signals within the PB as well as within the EB (Kakaria and de Bivort, 2017). The heading signal is mapped directly from visual signals in the ring neurons, which are subject to the position and orientation of the fly, and thus provide the egocentric (or body-centered) heading direction. However, an allocentric (or world-centric) heading signal is needed for the path integration process. Recent studies characterized a neuronal circuit that performs a series of vector calculations in the PB − fan-shaped body network based on translational visual cues to compute the allocentric traveling direction (Lu et al., 2021; Lyu et al., 2021). Finally, the polarization cue can be used for determining the flight direction (Weir and Dickinson, 2012; Hardcastle et al., 2021), and the underlying neural circuit has been dissected thoroughly, from the retina to the CX (Weir and Dickinson, 2012; Hardcastle et al., 2021).

## Visual Learning

**FIGURE 7:**
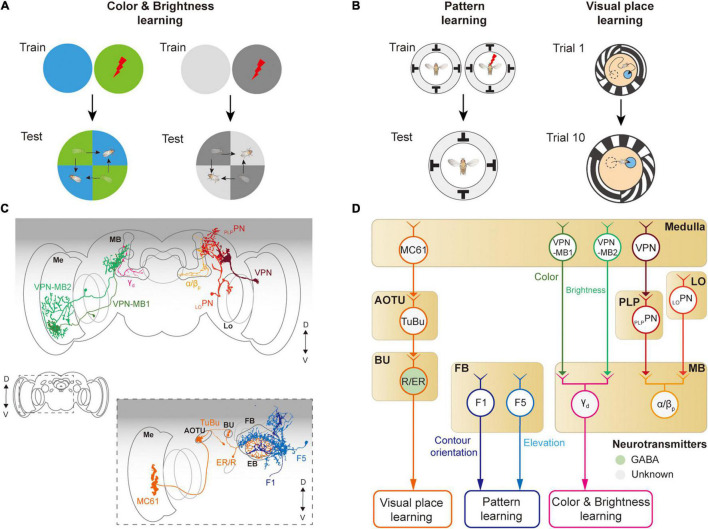
Neural circuits underlying visually learning. **(A)** When specific color or brightness were conditioned by a punitive stimulus, flies tend to avoid the conditioned visual feature. **(B)** When a flying or walking fly experiences heat shock for a specific visual pattern or landscape, flies learn to avoid the direction toward the pattern or remember the location of a cool spot. Each image of the behavioral setup depicts a top view of a cylindrical display arena. **(C)** Schematic illustration of neurons related to the visual learning. Graphical depiction is adapted from Vogt et al. (2016) and Li et al. (2020). The colors of neurons match those in panel **(D)**. Me: medulla, Lo: lobula, OG: optic glomeruli, AOTU: anterior optic tubercle, BU: bulb, PB: protocerebral bridge, EB: ellipsoid body, FB: fan-shaped body, MB: mushroom body. **(D)** Schematic diagram of neural circuits related to visual learning. The face color of a circle represents the type of neurotransmitters, and the line colors match those in panel **(C)**. OG: optic glomeruli, PLP: posterior lateral protocerebrum.

In 2012, Tomaso Poggio proposed to include learning as an additional layer to Marr’s three levels of analysis (Poggio, 2012), hence becoming four levels of analyses: learning, computation, algorithm, and implementation. *Drosophila* vision can be an excellent model system to study this additional layer, as flies are capable of associating various visual features with other sensory cues such as odor, food, temperature, and electric shock (Quinn et al., 1974; Guo and Guo, 2005; Ofstad et al., 2011; Ren et al., 2012; Aso et al., 2014; Vogt et al., 2014; Figures 7A,B). Developmental evidence suggested that both the MB and the CX are involved in visual learning (Barth and Heisenberg, 1997).

The first visual feature that was successfully conditioned in flies to other sensory stimuli was the color of the light (Quinn et al., 1974; Schnaitmann et al., 2010; Figure 7A). A brain structure essential for color learning is the MB, which is also a major structure for olfactory learning. The MB consists of five lobes (α, α′, β, β′, γ) and four calyces (one main and three accessory calyces) and receives the majority of olfactory information *via* the main calyx (Heisenberg et al., 1985; McGuire et al., 2001). Visual signals enter the MB *via* dorsal and ventral accessory calyces. Multiple types of visual projection neurons were identified to carry visual information from visual structures to the MB (Vogt et al., 2014; Li et al., 2020; Figure 7D). VPN-MB1 and VPN-MB2 neurons carry visual signals from the medulla to the MB, where they provide inputs to γd Kenyon cells (Vogt et al., 2016). _LO_PN and _PLP_PN carry inputs from the lobula and PVLP, respectively, to α/β_p_ Kenyon cells in the MB (Li et al., 2020; Figures 7C,D). VPN-MB1 was shown to be important for color learning, whereas VPN-MB2 was required for brightness learning (Vogt et al., 2016).

The MB is also required for learning visual patterns. If laser-heated for a specific visual pattern in an operant conditioning paradigm, tethered, flying *Drosophila* was shown to learn to avoid the direction of the visual pattern (Wolf and Heisenberg, 1991; Dill et al., 1993; Wolf et al., 1998; Figure 7B, left). However, mutant flies with the reduced size of MB were reported to have significantly impaired visual pattern learning (Liu et al., 1999; Tang and Guo, 2001). Other studies also suggested that the CX is involved in pattern learning. In particular, neurons in the fan-shaped body and ellipsoid body were shown to be critical for visual pattern learning (Liu et al., 2006; Pan et al., 2009). Place learning is another important behavior where visual pattern learning was demonstrated. In an arena consisting of heated blocks, flies learn the location of a cool, comfortable zone relative to the surrounding visual scene (Ofstad et al., 2011; Figure 7B, right). This spatial learning was shown to require a specific class of ring neurons, which carry visual information to the CX (Ofstad et al., 2011). These observations demonstrate that neurons in the MB as well as in the CX play an important role in visual associative memory formation for various visual features.

## Discussion

**FIGURE 8:**
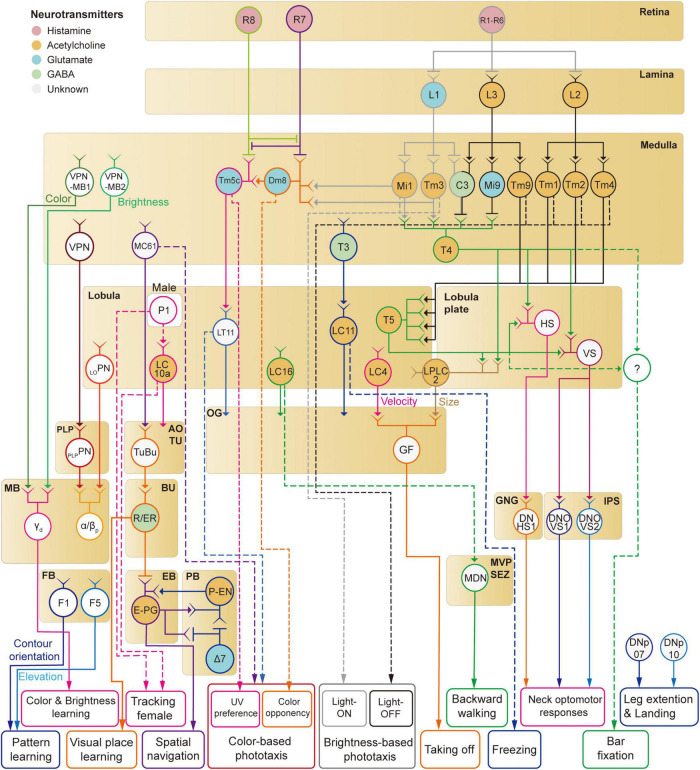
A schematic diagram of neural circuits involved in visual feature-based behaviors in *Drosophila*.

In this review, we have discussed neural implementations of visual behaviors in *Drosophila*, including phototaxis, optomotor response, object responses, navigation, and visual learning. We reviewed the visual circuits required for the detection of corresponding visual features (Figure 8). However, the understanding of the full visuomotor circuitry is still incomplete for most of these behaviors except a few cases such as the optomotor response.

Mammalian visual systems are well known to have two major visual pathways—dorsal and ventral streams—that represent different visual features. Is there a similar functional division in the *Drosophila* visual system? We have discussed so far at least four distinct visual projection pathways from the optic lobe to the central brain: (1) lobula plate to IPS/GNG, (2) lobula/lobula plate to optic glomeruli (3) medulla/lobula to AOTU, (4) medulla to the MB. First, the lobula-plate-to-IPS/GNG pathway seems to control reflexive behaviors such as optomotor response by directly signaling to descending neurons. Second, the optic glomeruli pathway consists of many different channels that appear to encode the shape and velocity of moving objects (Klapoetke et al., 2022). A subset of optic glomeruli connects directly to descending neurons for fast, reflexive actions, whereas the rest influences complex visual behaviors *via* less direct pathways. Third, whereas optic glomeruli have none or weak retinotopic information, the AOTU seems to represent the spatial information more faithfully than the optic glomeruli (Wu et al., 2016; Morimoto et al., 2020). It is this retinotopic information that is fed to the CX *via* the anterior visual pathway for the estimation of the position and orientation information. Overall, the AOTU pathway seems to be involved in innate visual behaviors such as courtship and navigation. Finally, the medulla-MB pathway is a key structure for visual learning in *Drosophila*. These observations suggest that visual signals enter different central brain structures with functional division matched to the function of each structure.

Our review is comprehensive but hardly complete. That is, some visual behaviors have not been discussed in case little is known about underlying neural circuits. An interesting visual behavior that has not been discussed is vision-based distant estimation. Walking flies are shown to be able to visually estimate the width of a gap and decide whether or not to attempt to cross the gap (Pick and Strauss, 2005; Triphan et al., 2016). Two cell types in the optic lobe were identified as related to this behavior, but it is unknown what visual features these neurons are sensitive to and how the gap-crossing behavior is controlled. Another example is found during the courtship behavior. During courtship, male flies vibrate their wings unilaterally to produce a courtship song whose amplitude is inversely proportional to the distance to the courted female, which suggests that male flies can visually estimate the distance from the female fly, but the neural circuit estimating the distance is so far unknown (Coen et al., 2016).

Visual responses of *Drosophila* are not fixed but can be flexibly modulated by the behavioral context, even for the same stimuli. For example, while walking or flying, the gain of motion-sensitive visual neurons increases, and the visual tuning shifts toward a higher motion velocity than the rest (Chiappe et al., 2010; Maimon et al., 2010). Furthermore, flies modulate their vision to distinguish between two types of whole-field visual motion: one caused by external events and the other by self-movement. Namely, motor-related inputs called efference copies can be sent to the visual system to selectively abolish self-motion-related visual feedback signals (von Holst and Mittelstaedt, 1950). Such motor-related inputs have been found in LPTCs (HS/VS cells), as well as in local interneurons in the OG (Kim et al., 2015). The amplitude of the motor-related inputs to HS/VS cells precisely matches the strength of the visual input in each cell type and changes depending on the ongoing visual drive (Kim A. J. et al., 2017). Finally, the motor-related inputs block the visual signaling during course-changing turns but not during course-stabilizing turns (Fenk et al., 2021). In walking flies, motor-related inputs arrive at HS/VS cells during turning, but their sign is in a direction that amplifies the visual feedback instead of suppressing it (Fujiwara et al., 2017). These studies highlight the possibility that *Drosophila* vision can be used to understand the complex interplay between the sensory system and the motor system in behaving animals.

Understanding information processing machines, such as computers and nervous systems, is a daunting endeavor considering their sheer complexity. As mentioned above, David Marr and Tomaso Poggio recommended that this venture be carried out at multiple levels in parallel, and the understanding from each level should be integrated to allow understanding of the whole (Marr and Poggio, 1976; Marr, 1985). The past two decades have seen substantial progress in understanding the functional and anatomical details underlying visual behaviors in *Drosophila*, that is, the implementation level of *Drosophila* vision. One important question is whether novel computations and algorithms can be newly discovered based on the discoveries at the implementation level. For example, studies on the detailed anatomy of the CX using sparse labeling or connectomic data have led to the formulation of detailed algorithms of how the structure maintains (Su et al., 2017) and moves the heading signal, which was later confirmed by experiments (Green et al., 2017). This is consistent with what Poggio emphasized as the synergy between the levels of analysis (Marr and Poggio, 1976; Marr, 1985). Furthermore, what we understand about the Drosophila vision at the computational and algorithmic levels is still limited. Recent opinion articles proposed to shift our attention toward characterizing behaviors in more naturalistic and unrestrained conditions (Datta et al., 2019; Pereira et al., 2020). Recent technological improvements in cinematography and artificial intelligence-based video analyses would allow detailed kinematic analyses, leading to novel visual behaviors (Stowers et al., 2017). Discovery of sophisticated behaviors in such conditions would stimulate studies on more complex brain functions in *Drosophila*.

## Author Contributions

LR and AJK originally conceived the project. LR, AJK, and SYK created the figures and wrote the manuscript. All authors contributed to the article and approved the submitted version.

## Conflict of Interest

The authors declare that the research was conducted in the absence of any commercial or financial relationships that could be construed as a potential conflict of interest.

## Publisher’s Note

All claims expressed in this article are solely those of the authors and do not necessarily represent those of their affiliated organizations, or those of the publisher, the editors and the reviewers. Any product that may be evaluated in this article, or claim that may be made by its manufacturer, is not guaranteed or endorsed by the publisher.

## References

[B1] Ache J. M. Namiki S. Lee A. Branson K. Card G. M. (2019a). State-dependent decoupling of sensory and motor circuits underlies behavioral flexibility in *Drosophila*. *Nat. Neurosci.* 22 1132–1139. 10.1038/s41593-019-0413-4 31182867 PMC7444277

[B2] Ache J. M. Polsky J. Alghailani S. Parekh R. Breads P. Peek M. Y. (2019b). Neural basis for looming size and velocity encoding in the *Drosophila* giant fiber escape pathway. *Curr. Biol* 29 1073–1081.e4. 10.1016/j.cub.2019.01.079 30827912

[B3] Agrawal S. Safarik S. Dickinson M. (2014). The relative roles of vision and chemosensation in mate recognition of *Drosophila melanogaster*. *J. Exp. Biol.* 217 2796–2805. 10.1242/jeb.105817 24902744

[B4] Aso Y. Sitaraman D. Ichinose T. Kaun K. R. Vogt K. Belliart-Guérin G. (2014). Mushroom body output neurons encode valence and guide memory-based action selection in *Drosophila*. *eLife* 3:e04580. 10.7554/eLife.04580 25535794 PMC4273436

[B5] Bacon J. P. Strausfeld N. J. (1986). The dipteran ‘Giant fibre’ pathway: neurons and signals. *J. Comp. Physiol.* 158 529–548. 10.1007/BF00603798

[B6] Bahl A. Ammer G. Schilling T. Borst A. (2013). Object tracking in motion-blind flies. *Nat. Neurosci.* 16 730–738. 10.1038/nn.3386 23624513

[B7] Barth M. Heisenberg M. (1997). Vision affects mushroom bodies and central complex in *Drosophila melanogaster*. *Learn. Mem.* 4 219–229. 10.1101/lm.4.2.219 10456065

[B8] Behbahani A. H. Palmer E. H. Corfas R. A. Dickinson M. H. (2021). *Drosophila* re-zero their path integrator at the center of a fictive food patch. *Curr. Biol.* 31 4534–4546.e5. 10.1016/j.cub.2021.08.006 34450090 PMC8551043

[B9] Behnia R. Clark D. A. Carter A. G. Clandinin T. R. Desplan C. (2014). Processing properties of ON and OFF pathways for *Drosophila* motion detection. *Nature* 512 427–430. 10.1038/nature13427 25043016 PMC4243710

[B10] Benzer S. (1967). Behavioral mutants of *Drosophila* isolated by countercurrent distribution. *Proc. Natl. Acad. Sci* 58 1112–1119. 10.1073/pnas.58.3.1112 16578662 PMC335755

[B11] Bertholf L. M. (1932). The extent of the spectrum for *Drosophila* and the distribution of stimulative efficiency in it. *Z. Vgl. Physiol.* 18 32–64. 10.1007/BF00338152

[B12] Bidaye S. S. Machacek C. Wu Y. Dickson B. J. (2014). Neuronal control of *Drosophila* walking direction. *Science* 344 97–101. 10.1126/science.1249964 24700860

[B13] Boergens K. M. Kapfer C. Helmstaedter M. Denk W. Borst A. (2018). Full reconstruction of large lobula plate tangential cells in *Drosophila* from a 3D EM dataset. *PLoS One* 13:e0207828. 10.1371/journal.pone.0207828 30485333 PMC6261601

[B14] Braitenberg V. (1967). Patterns of projection in the visual system of the fly. I. Retina-lamina projections. *Exp. Brain Res.* 3 271–298. 10.1007/BF00235589 6030825

[B15] Busch C. Borst A. Mauss A. S. (2018). Bi-directional control of walking behavior by horizontal optic flow sensors. *Curr. Biol* 28 4037–4045.e5. 10.1016/j.cub.2018.11.010 30528583

[B16] Chiappe M. E. Seelig J. D. Reiser M. B. Jayaraman V. (2010). Walking modulates speed sensitivity in *Drosophila* motion vision. *Curr. Biol.* 20 1470–1475. 10.1016/j.cub.2010.06.072 20655222 PMC4435946

[B17] Chou W. H. Hall K. J. Wilson D. B. Wideman C. L. Townson S. M. Chadwell L. V. (1996). Identification of a novel *Drosophila* opsin reveals specific patterning of the R7 and R8 photoreceptor cells. *Neuron* 17 1101–1115. 10.1016/S0896-6273(00)80243-3 8982159

[B18] Coen P. Xie M. Clemens J. Murthy M. (2016). Sensorimotor transformations underlying variability in song intensity during *Drosophila* courtship. *Neuron* 89 629–644. 10.1016/j.neuron.2015.12.035 26844835 PMC5047376

[B19] Datta S. R. Anderson D. J. Branson K. Perona P. Leifer A. (2019). Computational neuroethology: a call to action. *Neuron* 104 11–24. 10.1016/j.neuron.2019.09.038 31600508 PMC6981239

[B20] Davis F. P. Nern A. Picard S. Reiser M. B. Rubin G. M. Eddy S. R. (2020). A genetic, genomic, and computational resource for exploring neural circuit function. *eLife* 9:e50901. 10.7554/eLife.50901 31939737 PMC7034979

[B21] de Ruyter van Steveninck R. Bialek W. (1995). Reliability and statistical efficiency of a blowfly movement-sensitive neuron. *Philos. Trans. R. Soc. Lond. B Biol. Sci.* 348 321–340. 10.1098/rstb.1995.0071

[B22] Dill M. Wolf R. Heisenberg M. (1993). Visual pattern recognition in *Drosophila* involves retinotopic matching. *Nature* 365 751–753. 10.1038/365751a0 8413652

[B23] Feng K. Sen R. Minegishi R. Dübbert M. Bockemühl T. Büschges A. (2020). Distributed control of motor circuits for backward walking in *Drosophila*. *Nat. Commun.* 11:6166. 10.1038/s41467-020-19936-x 33268800 PMC7710706

[B24] Fenk L. M. Kim A. J. Maimon G. (2021). Suppression of motion vision during course-changing, but not course-stabilizing, navigational turns. *Curr. Biol.* 31 4608–4619.e3. 10.1016/j.cub.2021.09.068 34644548

[B25] Fenk L. M. Poehlmann A. Straw A. D. (2014). Asymmetric processing of visual motion for simultaneous object and background responses. *Curr. Biol.* 24 2913–2919. 10.1016/j.cub.2014.10.042 25454785

[B26] Ferreira C. H. Moita M. A. (2020). Behavioral and neuronal underpinnings of safety in numbers in fruit flies. *Nat. Commun.* 11:4182. 10.1038/s41467-020-17856-4 32826882 PMC7442810

[B27] Fischbach K. F. (1979). Simultaneous and successive colour contrast expressed in “slow” phototactic behaviour of walking *Drosophila melanogaster*. *J. Comp. Physiol.* 130 161–171. 10.1007/BF00611050

[B28] Fischbach K. F. Dittrich A. P. M. (1989). The optic lobe of *Drosophila melanogaster*. I. A Golgi analysis of wild-type structure. *Cell Tissue Res.* 258 441–475. 10.1007/BF00218858

[B29] Fisher Y. E. Leong J. C. Sporar K. Ketkar M. D. Gohl D. M. Clandinin T. R. (2015). A class of visual neurons with wide-field properties is required for local motion detection. *Curr. Biol.* 25 3178–3189. 10.1016/j.cub.2015.11.018 26670999

[B30] Fisher Y. E. Lu J. D’Alessandro I. Wilson R. I. (2019). Sensorimotor experience remaps visual input to a heading-direction network. *Nature* 576 121–125. 10.1038/s41586-019-1772-4 31748749 PMC7753972

[B31] Fortini M. E. Rubin G. M. (1990). Analysis of cis-acting requirements of the Rh3 and Rh4 genes reveals a bipartite organization to rhodopsin promoters in *Drosophila melanogaster*. *Genes Dev.* 4 444–463. 10.1101/gad.4.3.444 2140105

[B32] Franconville R. Beron C. Jayaraman V. (2018). Building a functional connectome of the *Drosophila* central complex. *eLife* 7:e37017. 10.7554/eLife.37017 30124430 PMC6150698

[B33] Fujiwara T. Cruz T. L. Bohnslav J. P. Chiappe M. E. (2017). A faithful internal representation of walking movements in the *Drosophila* visual system. *Nat. Neurosci.* 20 72–81. 10.1038/nn.4435 27798632

[B34] Gao S. Takemura S. Y. Ting C. Y. Huang S. Lu Z. Luan H. (2008). The neural substrate of spectral preference in *Drosophila*. *Neuron* 60 328–342. 10.1016/j.neuron.2008.08.010 18957224 PMC2665173

[B35] Gengs C. Leung H. T. Skingsley D. R. Iovchev M. I. Yin Z. Semenov E. P. (2002). The target of *Drosophila* photoreceptor synaptic transmission is a histamine-gated chloride channel encoded by ort (hclA). *J. Biol. Chem.* 277 42113–20. 10.1074/jbc.M207133200 12196539

[B36] Giraldo Y. M. Leitch K. J. Ros I. G. Warren T. L. Weir P. T. Dickinson M. H. (2018). Sun navigation requires compass neurons in *Drosophila*. *Curr. Biol.* 28 2845–2852.e4. 10.1016/j.cub.2018.07.002 30174187 PMC7301569

[B37] Götz K. G. (1964). Optomotorische Untersuchung des visuellen systems einiger Augenmutanten der Fruchtfliege *Drosophila*. *Kybernetik* 2 77–92. 10.1007/BF00288561 5833196

[B38] Götz K. G. (1970). Fractionation of *Drosophila* populations according to optomotor traits. *J. Exp. Biol.* 52 419–436. 10.1242/jeb.52.2.419 5442286

[B39] Green J. Adachi A. Shah K. K. Hirokawa J. D. Magani P. S. Maimon G. (2017). A neural circuit architecture for angular integration in *Drosophila*. *Nature* 546 101–106. 10.1038/nature22343 28538731 PMC6320684

[B40] Gruntman E. Romani S. Reiser M. B. (2018). Simple integration of fast excitation and offset, delayed inhibition computes directional selectivity in *Drosophila*. *Nat. Neurosci.* 21 250–257. 10.1038/s41593-017-0046-4 29311742 PMC5967973

[B41] Guo J. Guo A. (2005). Crossmodal Interactions Between Olfactory and Visual Learning in *Drosophila*. *Science* 309 307–310. 10.1126/science.1111280 16002621

[B42] Hadler N. M. (1964). Genetic influence on phototaxis in *Drosophila melanogaster*. *Biol. Bull.* 126 264–273. 10.2307/1539524 PMC1210733 14239787

[B43] Haikala V. Joesch M. Borst A. Mauss A. S. (2013). Optogenetic control of fly optomotor responses. *J. Neurosci.* 33 13927–13934. 10.1523/JNEUROSCI.0340-13.2013 23966712 PMC6618655

[B44] Hardcastle B. J. Omoto J. J. Kandimalla P. Nguyen B. C. M. Keleş M. F. Boyd N. K. (2021). A visual pathway for skylight polarization processing in *Drosophila*. *eLife* 10:e63225. 10.7554/eLife.63225 33755020 PMC8051946

[B45] Hardie R. C. (1985). Functional organization of the fly retina. *Prog. Sens. Physiol.* 5 1–79. 10.1007/978-3-642-70408-6_1

[B46] Hardie R. C. (1989). A histamine-activated chloride channel involved in neurotransmission at a photoreceptor synapse. *Nature* 339 704–706. 10.1038/339704a0 2472552

[B47] Hardie R. C. Raghu P. (2001). Visual transduction in *Drosophila*. *Nature* 413 186–193. 10.1038/35093002 11557987

[B48] Hassenstein B. (1951). Ommatidienraster und afferente Bewegungsintegration. *Z. Vgl. Physiol.* 33 301–326. 10.1007/BF00339334

[B49] Hassenstein B. Reichardt W. (1956). Systemtheoretische Analyse der Zeit-, Reihenfolgen- und Vorzeichenauswertung bei der Bewegungsperzeption des Rüsselkäfers Chlorophanus. *Z. Naturforsch. B J. Chem. Sci.* 11 513–524. 10.1515/znb-1956-9-1004

[B50] Hecht S. Wald G. (1934). The visual acuity and intensity discrimination of *Drosophila*. *J. Gen. Physiol.* 17 517–547. 10.1085/jgp.17.4.517 19872798 PMC2141300

[B51] Heisenberg M. Götz K. G. (1975). The use of mutations for the partial degradation of vision in *Drosophila melanogaster*. *J. Comp. Physiol.* 98 217–241. 10.1007/BF00656971

[B52] Heisenberg M. Wolf R. (1984). *Vision in *Drosophila*: Genetics of Microbehavior.* Berlin: Springer Verlag.

[B53] Heisenberg M. Borst A. Wagner S. Byers D. (1985). *Drosophila* mushroom body mutants are deficient in olfactory learning. *J. Neurogenet.* 2 1–30. 10.3109/01677068509100140 4020527

[B54] Heisenberg M. Wonneberger R. Wolf R. (1978). Optomotor-blindH31−a *Drosophila* mutant of the lobula plate giant neurons. *J. Comp. Physiol.* 124 287–296. 10.1007/BF00661379

[B55] Hu K. G. Stark W. S. (1977). Specific receptor input into spectral preference in *Drosophila*. *J. Comp. Physiol.* 121 241–252. 10.1007/BF00609614

[B56] Hulse B. K. Haberkern H. Franconville R. Turner-Evans D. B. Takemura S. Y. Wolff T. (2021). A connectome of the *Drosophila* central complex reveals network motifs suitable for flexible navigation and context-dependent action selection. *eLife* 10:e66039. 10.7554/eLife.66039 34696823 PMC9477501

[B57] Jenett A. Rubin G. M. Ngo T.-T. B. Shepherd D. Murphy C. Dionne H. (2012). A GAL4-Driver Line Resource for *Drosophila* Neurobiology. *Cell Reports* 2 991–1001. 10.1016/j.celrep.2012.09.011 23063364 PMC3515021

[B58] Joesch M. A. (2009). *Lobula Plate Tangential Cells in *Drosophila Melanogaster*; Response Properties, Synaptic Organization & Input Channels.* [Ph.D.thesis]. Munich, Germany: Ludwig Maximilian University of Munich.

[B59] Joesch M. Plett J. Borst A. Reiff D. F. (2008). Response properties of motion-sensitive visual interneurons in the lobula plate of *Drosophila melanogaster*. *Curr. Biol.* 18 368–374. 10.1016/j.cub.2008.02.022 18328703

[B60] Kakaria K. S. de Bivort B. L. (2017). Ring attractor dynamics emerge from a spiking model of the entire protocerebral bridge. *Front. Behav. Neurosci.* 11:8. 10.3389/fnbeh.2017.00008 28261066 PMC5306390

[B61] Kalmus H. (1943). The optomotor responses of some eye mutants of *Drosophila*. *J. Genet.* 45 206–213. 10.1007/BF02982936

[B62] Karuppudurai T. Lin T. Y. Ting C. Y. Pursley R. Melnattur K. V. Diao F. (2014). A hard-wired glutamatergic circuit pools and relays UV signals to mediate spectral preference in *Drosophila*. *Neuron* 81 603–615. 10.1016/j.neuron.2013.12.010 24507194 PMC3920195

[B63] Katayama N. Abbott J. K. Kjærandsen J. Takahashi Y. Svensson E. I. (2014). Sexual selection on wing interference patterns in *Drosophila melanogaster*. *Proc. Natl. Acad. Sci.* 111 15144–15148. 10.1073/pnas.1407595111 25294931 PMC4210313

[B64] Keleş M. F. Frye M. A. (2017). Object-detecting neurons in *Drosophila*. *Curr. Biol.* 27 680–687. 10.1016/j.cub.2017.01.012 28190726 PMC5340600

[B65] Ketkar M. D. Sporar K. Gür B. Ramos-Traslosheros G. Seifert M. Silies M. (2020). Luminance information is required for the accurate estimation of contrast in rapidly changing visual contexts. *Curr. Biol.* 30 657–669.e4. 10.1016/j.cub.2019.12.038 32008904

[B66] Kim A. J. Fenk L. M. Lyu C. Maimon G. (2017). Quantitative predictions orchestrate visual signaling in *Drosophila*. *Cell* 168 280–294.e12. 10.1016/j.cell.2016.12.005 28065412 PMC6320683

[B67] Kim A. J. Fitzgerald J. K. Maimon G. (2015). Cellular evidence for efference copy in *Drosophila* visuomotor processing. *Nat. Neurosci.* 18 1247–1255. 10.1038/nn.4083 26237362 PMC6327952

[B68] Kim I. S. Dickinson M. H. (2017). Idiothetic path integration in the fruit fly *Drosophila melanogaster*. *Curr. Biol.* 27 2227–2238. 10.1016/j.cub.2017.06.026 28736164

[B69] Kim S. S. Hermundstad A. M. Romani S. Abbott L. F. Jayaraman V. (2019). Generation of stable heading representations in diverse visual scenes. *Nature* 576 126–131. 10.1038/s41586-019-1767-1 31748750 PMC8115876

[B70] Kim S. S. Rouault H. Druckmann S. Jayaraman V. (2017). Ring attractor dynamics in the *Drosophila* central brain. *Science* 356 849–853. 10.1126/science.aal4835 28473639

[B71] Kirschfeld K. Franceschini N. Minke B. (1977). Evidence for a sensitising pigment in fly photoreceptors. *Nature* 269 386–390. 10.1038/269386a0 909585

[B72] Klapoetke N. C. Nern A. Peek M. Y. Rogers E. M. Breads P. Rubin G. M. (2017). Ultra-selective looming detection from radial motion opponency. *Nature* 551 237–241. 10.1038/nature24626 29120418 PMC7457385

[B73] Klapoetke N. C. Nern A. Rogers E. M. Rubin G. M. Reiser M. B. Card G. M. (2022). A functionally ordered visual feature map in the *Drosophila* brain. *Neuron* 110 1–12. 10.1016/j.neuron.2022.02.013 35290791

[B74] Kohatsu S. Yamamoto D. (2015). Visually induced initiation of *Drosophila* innate courtship-like following pursuit is mediated by central excitatory state. *Nat. Commun.* 6:6457. 10.1038/ncomms7457 25743851

[B75] Krapp H. G. Hengstenberg B. Hengstenberg R. (1998). Dendritic structure and receptive-field organization of optic flow processing interneurons in the fly. *J. Neurophysiol.* 79 1902–1917. 10.1152/jn.1998.79.4.1902 9535957

[B76] Lazopulo S. Lazopulo A. Baker J. D. Syed S. (2019). Daytime colour preference in *Drosophila* depends on the circadian clock and TRP channels. *Nature* 574 108–111. 10.1038/s41586-019-1571-y 31534223

[B77] Li J. Mahoney B. D. Jacob M. S. Caron S. J. C. (2020). Visual input into the *Drosophila melanogaster* mushroom body. *Cell Rep.* 32:108138. 10.1016/j.celrep.2020.108138 32937130 PMC8252886

[B78] Li Y. Chen P. J. Lin T. Y. Ting C. Y. Muthuirulan P. Pursley R. (2021). Neural mechanism of spatio-chromatic opponency in the *Drosophila* amacrine neurons. *Curr. Biol.* 31 3040–3052.e9. 10.1016/j.cub.2021.04.068 34033749

[B79] Lin T. Y. Luo J. Shinomiya K. Ting C. Y. Lu Z. Meinertzhagen I. A. (2016). Mapping chromatic pathways in the *Drosophila* visual system. *J. Comp. Neurol.* 524 213–227. 10.1002/cne.23857 26179639 PMC4678965

[B80] Liu G. Seiler H. Wen A. Zars T. Ito K. Wolf R. (2006). Distinct memory traces for two visual features in the *Drosophila* brain. *Nature* 439 551–556. 10.1038/nature04381 16452971

[B81] Liu L. Wolf R. Ernst R. Heisenberg M. (1999). Context generalization in *Drosophila* visual learning requires the mushroom bodies. *Nature* 400 753–756. 10.1038/23456 10466722

[B82] Lu J. Behbahani A. H. Hamburg L. Westeinde E. A. Dawson P. M. Lyu C. (2021). Transforming representations of movement from body- to world-centric space. *Nature* 601 98–104. 10.1038/s41586-021-04191-x 34912123 PMC10759448

[B83] Lyu C. Abbott L. F. Maimon G. (2021). Building an allocentric travelling direction signal via vector computation. *Nature* 601 92–97. 10.1038/s41586-021-04067-0 34912112 PMC11104186

[B84] Maimon G. Straw A. D. Dickinson M. H. (2008). A simple vision-based algorithm for decision making in flying *Drosophila*. *Curr. Biol.* 18 464–470. 10.1016/j.cub.2008.02.054 18342508

[B85] Maimon G. Straw A. D. Dickinson M. H. (2010). Active flight increases the gain of visual motion processing in *Drosophila*. *Nat. Neurosci.* 13 393–399. 10.1038/nn.2492 20154683

[B86] Maisak M. S. Haag J. Ammer G. Serbe E. Meier M. Leonhardt A. (2013). A directional tuning map of *Drosophila* elementary motion detectors. *Nature* 500 212–216. 10.1038/nature12320 23925246

[B87] Marr D. (1985). *Vision: A Computational Investigation into the Human Representation and Processing of Visual Information.* Massachusetts: MIT Press.

[B88] Marr D. Poggio T. (1976). *From Understanding Computation to Understanding Neural Circuitry. AI Memos AIM-357.* Massachusetts: MIT.

[B89] Mauss A. S. Borst A. (2020). Optic flow-based course control in insects. *Curr. Opin. Neurobiol.* 60 21–27. 10.1016/j.conb.2019.10.007 31810007

[B90] Mauss A. S. Meier M. Serbe E. Borst A. (2014). Optogenetic and pharmacologic dissection of feedforward inhibition in *Drosophila* motion vision. *J. Neurosci.* 34 2254–2263. 10.1523/JNEUROSCI.3938-13.2014 24501364 PMC6608528

[B91] Mauss A. S. Pankova K. Arenz A. Nern A. Rubin G. M. Borst A. (2015). Neural circuit to integrate opposing motions in the visual field. *Cell* 162 351–362. 10.1016/j.cell.2015.06.035 26186189

[B92] McGuire S. E. Le P. T. Davis R. L. (2001). The role of *Drosophila* mushroom body signaling in olfactory memory. *Science* 293 1330–1333. 10.1126/science.1062622 11397912

[B93] Meinertzhagen I. A. O’Neil S. D. (1991). Synaptic organization of columnar elements in the lamina of the wild type in *Drosophila melanogaster*. *J. Comp. Neurol* 305 232–63. 10.1002/cne.903050206 1902848

[B94] Molina-Obando S. Vargas-Fique J. F. Henning M. Gür B. Schladt T. M. Akhtar J. (2019). ON selectivity in the *Drosophila* visual system is a multisynaptic process involving both glutamatergic and GABAergic inhibition. *eLife* 8:e49373. 10.7554/elife.49373 31535971 PMC6845231

[B95] Montell C. Jones K. Zuker C. Rubin G. (1987). A second opsin gene expressed in the ultraviolet-sensitive R7 photoreceptor cells of *Drosophila melanogaster*. *J. Neurosci.* 7 1558–1566. 10.1523/JNEUROSCI.07-05-01558.1987 2952772 PMC6568825

[B96] Morimoto M. M. Nern A. Zhao A. Rogers E. M. Wong A. M. Isaacson M. D. (2020). Spatial readout of visual looming in the central brain of *Drosophila*. *eLife* 9:e57685. 10.7554/eLife.57685 33205753 PMC7744102

[B97] Muijres F. T. Elzinga M. J. Melis J. M. Dickinson M. H. (2014). Flies evade looming targets by executing rapid visually directed banked turns. *Science* 344 172–177. 10.1126/science.1248955 24723606

[B98] Namiki S. Dickinson M. H. Wong A. M. Korff W. Card G. M. (2018). The functional organization of descending sensory-motor pathways in *Drosophila*. *eLife* 7:e34272. 10.7554/elife.34272 29943730 PMC6019073

[B99] Ofstad T. A. Zuker C. S. Reiser M. B. (2011). Visual place learning in *Drosophila melanogaster*. *Nature* 474 204–207. 10.1038/nature10131 21654803 PMC3169673

[B100] Omoto J. J. Keleş M. F. Nguyen B.-C. M. Bolanos C. Lovick J. K. Frye M. A. (2017). Visual Input to the *Drosophila* Central Complex by Developmentally and Functionally Distinct Neuronal Populations. *Curr. Biol.* 27 1098–1110. 10.1016/j.cub.2017.02.063 28366740 PMC5446208

[B101] O’Tousa J. E. Baehr W. Martin R. L. Hirsh J. Pak W. L. Applebury M. L. (1985). The *Drosophila* ninaE gene encodes an opsin. *Cell* 40 839–850. 10.1016/0092-8674(85)90343-5 2985266

[B102] Otsuna H. Ito K. (2006). Systematic analysis of the visual projection neurons of *Drosophila melanogaster*. I. Lobula-specific pathways. *J. Comp. Neurol.* 497 928–958. 10.1002/cne.21015 16802334

[B103] Otsuna H. Shinomiya K. Ito K. (2014). Parallel neural pathways in higher visual centers of the *Drosophila* brain that mediate wavelength-specific behavior. *Front. Neural Circuits* 8:8. 10.3389/fncir.2014.00008 24574974 PMC3918591

[B104] Pagni M. Haikala V. Oberhauser V. Meyer P. B. Reiff D. F. Schnaitmann C. (2021). Interaction of “chromatic” and “achromatic” circuits in *Drosophila* color opponent processing. *Curr. Biol.* 31 1687–1698.e4. 10.1016/j.cub.2021.01.105 33636123

[B105] Pan Y. Zhou Y. Guo C. Gong H. Gong Z. Liu L. (2009). Differential roles of the fan-shaped body and the ellipsoid body in *Drosophila* visual pattern memory. *Learn. Mem.* 16 289–295. 10.1101/lm.1331809 19389914

[B106] Panser K. Tirian L. Schulze F. Villalba S. Jefferis G. S. Bühler K. (2016). Automatic segmentation of *Drosophila* neural compartments using GAL4 expression data reveals novel visual pathways. *Curr. Biol.* 26 1943–1954. 10.1016/j.cub.2016.05.052 27426516 PMC4985560

[B107] Papatsenko D. Sheng G. Desplan C. (1997). A new rhodopsin in R8 photoreceptors of *Drosophila*: evidence for coordinate expression with Rh3 in R7 cells. *Development* 124 1665–1673. 10.1242/dev.124.9.1665 9165115

[B108] Pereira T. D. Shaevitz J. W. Murthy M. (2020). Quantifying behavior to understand the brain. *Nat. Neurosci.* 23 1537–1549. 10.1038/s41593-020-00734-z 33169033 PMC7780298

[B109] Pick S. Strauss R. (2005). Goal-driven behavioral adaptations in gap-climbing *Drosophila*. *Curr. Biol.* 15 1473–1478. 10.1016/j.cub.2005.07.022 16111941

[B110] Poggio T. (2012). The levels of understanding framework, revised. *Perception* 41 1017–1023. 10.1068/p7299 23409366

[B111] Quinn W. G. Harris W. A. Benzer S. (1974). Conditioned Behavior in *Drosophila melanogaster*. *Proc. Natl. Acad. Sci. U.S.A.* 71 708–712. 10.1073/pnas.71.3.708 4207071 PMC388082

[B112] Raji J. I. Potter C. J. (2021). The number of neurons in *Drosophila* and mosquito brains. *PLoS One* 16:e0250381. 10.1371/journal.pone.0250381 33989293 PMC8121336

[B113] Ramos-Traslosheros G. Silies M. (2021). The physiological basis for contrast opponency in motion computation in *Drosophila*. *Nat. Commun.* 12:4987. 10.1038/s41467-021-24986-w 34404776 PMC8371135

[B114] Reichardt W. Poggio T. (1975). A theory of the pattern induced flight orientation of the fly *Musca domestica* II. *Biol. Cybern.* 18 69–80. 10.1007/BF00337127 1138981

[B115] Reichardt W. Poggio T. (1976). Visual control of orientation behaviour in the fly. Part I. A quantitative analysis. *Q. Rev. Biophys.* 9 311–75. 10.1017/S0033583500002523 790441

[B116] Reichardt W. Wenking H. (1969). Optical detection and fixation of objects by fixed flying flies. *Naturwissenschaften* 56 424–425. 10.1007/bf00593644 5362004

[B117] Ren Q. Li H. Wu Y. Ren J. Guo A. (2012). A GABAergic Inhibitory Neural Circuit Regulates Visual Reversal Learning in *Drosophila*. *J. Neurosci.* 32 11524–11538. 10.1523/JNEUROSCI.0827-12.2012 22915099 PMC6703766

[B118] Ribeiro I. M. A. Drews M. Bahl A. Machacek C. Borst A. Dickson B. J. (2018). Visual projection neurons mediating directed courtship in *Drosophila*. *Cell* 174 607–621.e18. 10.1016/j.cell.2018.06.020 30033367

[B119] Rister J. Desplan C. (2011). The retinal mosaics of opsin expression in invertebrates and vertebrates. *Dev. Neurobiol.* 71 1212–1226. 10.1002/dneu.20905 21557510 PMC3190030

[B120] Rister J. Pauls D. Schnell B. Ting C. Y. Lee C. H. Sinakevitch I. (2007). Dissection of the peripheral motion channel in the visual system of *Drosophila melanogaster*. *Neuron* 56 155–170. 10.1016/j.neuron.2007.09.014 17920022

[B121] Salcedo E. Huber A. Henrich S. Chadwell L. V. Chou W. H. (1999). Blue- and green-absorbing visual pigments of *Drosophila*: ectopic expression and physiological characterization of the R8 photoreceptor cell-specific Rh5 and Rh6 rhodopsins. *J. Neurosci.* 19 10716–10726. 10.1523/JNEUROSCI.1999 10594055 PMC6784940

[B122] Scheffer L. K. Xu C. S. Januszewski M. Lu Z. Takemura S. Y. Hayworth K. J. (2020). A connectome and analysis of the adult *Drosophila* central brain. *eLife* 9:e57443. 10.7554/eLife.57443 32880371 PMC7546738

[B123] Schnaitmann C. Haikala V. Abraham E. Oberhauser V. Thestrup T. Griesbeck O. (2018). Color processing in the early visual system of *Drosophila*. *Cell* 172 318–330.e18. 10.1016/j.cell.2017.12.018 29328919

[B124] Schnaitmann C. Vogt K. Triphan T. Tanimoto H. (2010). Appetitive and aversive visual learning in freely moving *Drosophila*. *Front. Behav. Neurosci.* 4:10. 10.3389/fnbeh.2010.00010 20300462 PMC2839846

[B125] Schnell B. Joesch M. Forstner F. Raghu S. V. Otsuna H. Ito K. (2010). Processing of horizontal optic flow in three visual interneurons of the *Drosophila* brain. *J. Neurophysiol.* 103 1646–1657. 10.1152/jn.00950.2009 20089816

[B126] Schümperli R. A. (1973). Evidence for colour vision in *Drosophila melanogaster* through spontaneous phototactic choice behaviour. *J. Comp. Physiol. A* 86 77–94. 10.1007/BF00694480

[B127] Seelig J. D. Jayaraman V. (2013). Feature detection and orientation tuning in the *Drosophila* central complex. *Nature* 503 262–266. 10.1038/nature12601 24107996 PMC3830704

[B128] Seelig J. D. Jayaraman V. (2015). Neural dynamics for landmark orientation and angular path integration. *Nature* 521 186–191. 10.1038/nature14446 25971509 PMC4704792

[B129] Sen R. Wu M. Branson K. Robie A. Rubin G. M. Dickson B. J. (2017). Moonwalker descending neurons mediate visually evoked retreat in *Drosophila*. *Curr. Biol.* 27 766–771. 10.1016/j.cub.2017.02.008 28238656

[B130] Sherman A. Dickinson M. H. (2004). Summation of visual and mechanosensory feedback in *Drosophila* flight control. *J. Exp. Biol.* 207 133–142. 10.1242/jeb.00731 14638840

[B131] Shinomiya K. Huang G. Lu Z. Parag T. Xu C. S. Aniceto R. (2019). Comparisons between the ON- and OFF-edge motion pathways in the *Drosophila* brain. *eLife* 8:e40025. 10.7554/eLife.40025 30624205 PMC6338461

[B132] Silies M. Gohl D. M. Fisher Y. E. Freifeld L. Clark D. A. Clandinin T. R. (2013). Modular use of peripheral input channels tunes motion-detecting circuitry. *Neuron* 79 111–127. 10.1016/j.neuron.2013.04.029 23849199 PMC3713415

[B133] Städele C. Keleş M. F. Mongeau J. M. Frye M. A. (2020). Non-canonical receptive field properties and neuromodulation of feature-detecting neurons in flies. *Curr. Biol.* 30 2508–2519. 10.1016/j.cub.2020.04.069 32442460 PMC7343589

[B134] Stark W. S. Walker J. A. Harris W. A. (1976). Genetic dissection of the photoreceptor system in the compound eye of *Drosophila melanogaster*. *J. Physiol.* 256 415–439. 10.1113/jphysiol.1976.sp011331 16992509 PMC1309314

[B135] Sten T. H. Li R. Otopalik A. Ruta V. (2021). Sexual arousal gates visual processing during *Drosophila* courtship. *Nature* 595 549–553. 10.1038/s41586-021-03714-w 34234348 PMC8973426

[B136] Stowers J. R. Hofbauer M. Bastien R. Griessner J. Higgins P. Farooqui S. (2017). Virtual reality for freely moving animals. *Nat. Methods* 14 995–1002. 10.1038/nmeth.4399 28825703 PMC6485657

[B137] Strother J. A. Nern A. Reiser M. B. (2014). Direct observation of ON and OFF pathways in the *Drosophila* visual system. *Curr. Biol.* 24 976–983. 10.1016/j.cub.2014.03.017 24704075

[B138] Strother J. A. Wu S. T. Wong A. M. Nern A. Rogers E. M. Le J. Q. (2017). The emergence of directional selectivity in the visual motion pathway of *Drosophila*. *Neuron* 94 168–182.e10. 10.1016/j.neuron.2017.03.010 28384470

[B139] Su T. S. Lee W. J. Huang Y. C. Wang C. T. Lo C. C. (2017). Coupled symmetric and asymmetric circuits underlying spatial orientation in fruit flies. *Nat. Commun.* 8:139. 10.1038/s41467-017-00191-6 28747622 PMC5529380

[B140] Suver M. P. Huda A. Iwasaki N. Safarik S. Dickinson M. H. (2016). An array of descending visual interneurons encoding self-motion in *Drosophila*. *J. Neurosci.* 36 11768–11780. 10.1523/JNEUROSCI.2277-16.2016 27852783 PMC5125229

[B141] Takemura S. Y. Bharioke A. Lu Z. Nern A. Vitaladevuni S. Rivlin P. K. (2013). A visual motion detection circuit suggested by *Drosophila* connectomics. *Nature* 500 175–181. 10.1038/nature1245 23925240 PMC3799980

[B142] Tanaka R. Clark D. A. (2020). Object-displacement-sensitive visual neurons drive freezing in *Drosophila*. *Curr. Biol.* 30 2532–2550. 10.1016/j.cub.2020.04.068 32442466 PMC8716191

[B143] Tang S. Guo A. (2001). Choice behavior of *Drosophila* facing contradictory visual cues. *Science* 294 1543–1547. 10.1126/science.1058237 11711680

[B144] Timaeus L. Geid L. Sancer G. Wernet M. F. Hummel T. (2020). Coupled symmetric and asymmetric circuits underlying spatial orientation in fruit flies. *iScience* 23:101590. 10.1016/j.isci.2020.101590 33205011 PMC7648135

[B145] Triphan T. Nern A. Roberts S. F. Korff W. Naiman D. Q. Strauss R. (2016). A screen for constituents of motor control and decision making in *Drosophila* reveals visual distance-estimation neurons. *Sci. Rep.* 6:27000. 10.1038/srep27000 27255169 PMC4891706

[B146] Turner-Evans D. B. Jensen K. T. Ali S. Paterson T. Sheridan A. Ray R. P. (2020). The neuroanatomical ultrastructure and function of a biological ring attractor. *Neuron* 108 145–163.e10. 10.1016/j.neuron.2020.08.006 32916090 PMC8356802

[B147] van Breugel F. Dickinson M. H. (2012). The visual control of landing and obstacle avoidance in the fruit fly *Drosophila melanogaster*. *J. Exp. Biol.* 215 1783–1798. 10.1242/jeb.066498 22573757

[B148] Vogt K. Aso Y. Hige T. Knapek S. Ichinose T. Friedrich A. B. (2016). Direct neural pathways convey distinct visual information to *Drosophila* mushroom bodies. *eLife* 5:e14009. 10.7554/eLife.14009 27083044 PMC4884080

[B149] Vogt K. Schnaitmann C. Dylla K. V. Knapek S. Aso Y. Rubin G. M. (2014). Shared mushroom body circuits underlie visual and olfactory memories in *Drosophila*. *eLife* 3:e02395. 10.7554/eLife.02395 25139953 PMC4135349

[B150] von Holst E. Mittelstaedt H. (1950). Das Reafferenzprinzip: Wechselwirkungen zwischen Zentralnervensystem und Peripherie. *Naturwissenschaften* 37 464–476. 10.1007/BF00622503

[B151] von Reyn C. R. Breads P. Peek M. Y. Zheng G. Z. Williamson W. R. Yee A. L. (2014). A spike-timing mechanism for action selection. *Nat. Neurosci.* 17 962–970. 10.1038/nn.3741 24908103

[B152] Wardill T. J. List O. Li X. Dongre S. McCulloch M. Ting C. Y. (2012). Multiple spectral inputs improve motion discrimination in the *Drosophila* visual system. *Science* 336 925–931. 10.1126/science.1215317 22605779 PMC6528803

[B153] Warren T. L. Weir P. T. Dickinson M. H. (2018). Flying *Drosophila melanogaster* maintain arbitrary but stable headings relative to the angle of polarized light. *J. Exp. Biol.* 221:jeb177550. 10.1242/jeb.177550 29593084

[B154] Weir P. T. Dickinson M. H. (2012). Flying *Drosophila* orient to sky polarization. *Curr. Biol.* 22 21–27. 10.1016/j.cub.2011.11.026 22177905 PMC4641755

[B155] Wolf R. Heisenberg M. (1991). Basic organization of operant behavior as revealed in *Drosophila* flight orientation. *J. Comp. Physiol. A* 169 699–705. 10.1007/BF00194898 1795235

[B156] Wolf R. Wittig T. Liu L. Wustmann G. Eyding D. Heisenberg M. (1998). *Drosophila* Mushroom Bodies Are Dispensable for Visual, Tactile, and Motor Learning. *Learn. Mem.* 5 166–178. 10.1101/lm.5.1.166 10454381 PMC311233

[B157] Wolff T. Iyer N. A. Rubin G. M. (2014). Neuroarchitecture and neuroanatomy of the *Drosophila* central complex: a GAL4-based dissection of protocerebral bridge neurons and circuits. *J. Comp. Neurol.* 523 997–1037. 10.1002/cne.23705 25380328 PMC4407839

[B158] Wu M. Nern A. Williamson W. R. Morimoto M. M. Reiser M. B. Card G. M. (2016). Visual projection neurons in the *Drosophila* lobula link feature detection to distinct behavioral programs. *eLife* 5:e21022. 10.7554/eLife.21022 28029094 PMC5293491

[B159] Xie X. Tabuchi M. Brown M. P. Mitchell S. P. Wu M. N. Kolodkin A. L. (2017). The laminar organization of the *Drosophila* ellipsoid body is semaphorin-dependent and prevents the formation of ectopic synaptic connections. *eLife* 6:e25328. 10.7554/eLife.25328 28632130 PMC5511011

[B160] Yamaguchi S. Desplan C. Heisenberg M. (2010). Contribution of photoreceptor subtypes to spectral wavelength preference in *Drosophila*. *Proc. Natl. Acad. Sci.* 107 5634–5639. 10.1073/pnas.0809398107 20212139 PMC2851746

[B161] Yang H. H. St-Pierre F. Sun X. Ding X. Lin M. Z. Clandinin T. R. (2016). Subcellular imaging of voltage and calcium signals reveals neural processing in vivo. *Cell* 166 245–257. 10.1016/j.cell.2016.05.031 27264607 PMC5606228

[B162] Zheng Z. Lauritzen J. S. Perlman E. Robinson C. G. Nichols M. Milkie D. (2018). A complete electron microscopy volume of the brain of adult *Drosophila melanogaster*. *Cell* 174 730–743.e22. 10.1016/j.cell.2018.06.019 30033368 PMC6063995

